# Low-Rank Adaptation of Pre-Trained Large Vision Models for Improved Lung Nodule Malignancy Classification

**DOI:** 10.1109/OJEMB.2025.3530841

**Published:** 2025-01-16

**Authors:** Benjamin P. Veasey, Amir A. Amini

**Affiliations:** Medical Imaging LaboratoryUniversity of Louisville 5170 Louisville KY 40208 USA

**Keywords:** Low-rank adaptation, lung cancer, nodule classification, parameter-efficient fine-tuning, vision transformers

## Abstract

*Goal:* This paper investigates using Low-Rank Adaptation (LoRA) to adapt large vision models (LVMs) pretrained with self-supervised learning (SSL) for lung nodule malignancy classification. Inspired by LoRA's success in the field of Natural Language Processing, we hypothesized that such an adaptation technique can significantly improve classification performance, parameter efficiency, and training speed for the novel application of lung image cancer diagnostic. *Methods:* Utilizing two comprehensive lung nodule datasets, NLSTx and LIDC, which together encompass a diverse array of biopsy- and radiologist-confirmed lung CT scans, our rigorous experimental setup demonstrates that LoRA-adapted models markedly surpass traditional fine-tuning methods. *Results:* The best LoRA-adapted model achieved a 3% increase in ROC AUC over the state-of-the-art model, utilized 89.9% fewer parameters, and reduced training times by 36.5%. *Conclusions:* Integrating LoRA with out-of-domain pretrained LVMs offers a promising avenue for enhancing performance of lung nodule malignancy classification. The annotations for the NLSTx dataset are also released with this paper on GitHub at https://github.com/benVZ/NLSTx.

## I. Introduction

Training classifiers for lung nodule malignancy from the ground up is fraught with challenges: it's a slow process that demands extensive datasets for optimal performance and typically relies on neural networks with numerous parameters.

In the realm of natural images, self-supervised learning (SSL) has made recent strides, enabling the creation of foundational image models that serve as efficient feature extractors for subsequent classifier training. Translating these successes to the lung cancer domain has proven difficult, largely due to stringent data protection laws and the absence of organizations capable of amassing extensive thoracic CT images. Consequently, there is a scarcity of large, publicly available lung cancer imaging datasets. For instance, the National Lung Screening Trials (NLST) [14] managed to collect around 50,000 unique patient data, a feat not commonly seen in medical imaging data collection efforts. The MedSAM [12] dataset curated 1.57 million open-source images but less than 500 are lung CT scans while RadImageNet [15] boasts 1.35 million images, with 152000 lung CT scans – the largest to date. However, these numbers pale in comparison to the vast datasets in the natural image domain, essential for advancing SSL-trained large vision models (LVMs). Despite their heterogeneity, multi-institutional medical imaging trials often do not reflect the full diversity of images from different scan manufacturers, resolutions, image contrast, and acquisition techniques regardless of standardized protocols.

Over the past ten years, two main deep learning strategies have emerged for lung cancer classification: either accumulate sufficient data to train a model from scratch, accepting inherent limitations, or fine-tune a network [8] initially trained on large-scale natural image datasets.

The traditional fine-tuning method involves further training some or all of the pretrained network's layers on a new lung cancer dataset at a reduced learning rate. Properly executed, this approach can yield a network with high classification accuracy for lung cancer. However, this method has significant downsides. It requires many trainable parameters, which can increase the GPU memory footprint, potentially lengthening training times and using considerable disk space – for instance, large Vision Transformers (ViT) [5] can require gigabytes to store their network weights after training is complete.

To address these challenges, a promising approach known as Parameter-Efficient Fine-Tuning (PEFT), which has gained popularity in Natural Language Processing (NLP) research, is garnering attention. Our current study, an extension of our previous work [20] – investigates the use of a PEFT method, Low Rank Adaptation (LoRA) [7], to evaluate its effectiveness in adapting high-performing self-supervised learning (SSL) pretrained ViT [5] and Swin [11] transformers for lung cancer malignancy classification. Building on successes observed in NLP, we hypothesized that applying LoRA to adapt these LVMs on two lung nodule datasets, NLSTx [18] and LIDC [1], would yield several advantages over traditionally fine-tuned LVMs and Convolutional Neural Networks (CNNs), including:

1) Enhanced lung cancer classification accuracy,
2) Improved parameter efficiency,
3) Reduced training time

**Fig. 1.**
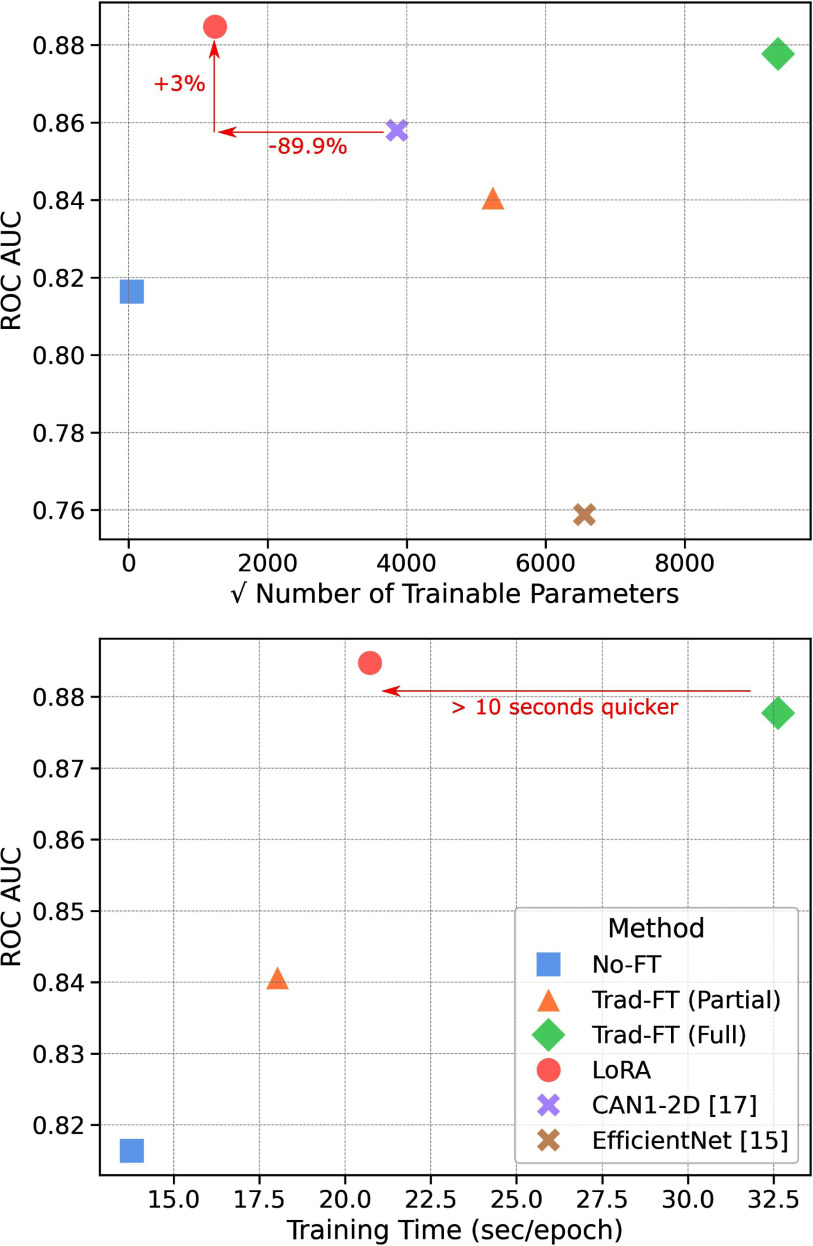
Plots showing that LoRA tuning of SwinV2-b (red circle) beats the previous state-of-the-art (purple X) on NLSTx by 3% ROC AUC with an 89.9% reduction in parameters while achieving higher classification performance, using significantly fewer trainable parameters, and training quicker than traditional fine-tuning of transformers. No-FT = No Fine-tuning, trad-FT (Partial) = partial parameter fine-tuning (CNNs only), trad-FT (Full) = full parameter fine-tuning.

A comprehensive statistical analysis revealed that the LoRA-adapted LVMs outperformed the state-of-the-art method by up to 3% ROC AUC, while utilizing 89.9% fewer trainable parameters (see Fig. 1). Additionally, these models performed comparably to traditionally fine-tuned models, while utilizing a remarkable 85 million fewer trainable parameters and a 36.5% decrease in training time per iteration. Although fine-tuning a large CNN model, ConvNext-V2 [21], resulted in faster training times, the best LoRA-adapted transformers significantly outperformed the fine-tuned CNN in classification accuracy, all while requiring far fewer trainable parameters. This highlights a practical approach for deploying transformer-based LVMs in lung cancer diagnostics. The subsequent sections provide a detailed account of the experimental procedures and findings.

The key contributions of this paper are as follows:

1) Utilizing LoRA parameter-efficient fine-tuning to adapt and train several state-of-the-art large vision models, pretrained on natural images, for lung malignancy classification. A broad range of possibilities was explored, encompassing various input image configurations, model architectures, and adaptation techniques.
2) Conducting a rigorous analysis to identify emerging patterns across the various combinations.
3) Validating all methods and combinations against prior state-of-the-art methods on two independent lung cancer datasets.
4) Releasing the NLSTx [18] data for general use at the time of this paper's publication to GitHub. This data includes bounding box annotations for corresponding nodules in successive time points, with 647 biopsy-confirmed nodules (263 malignant and 384 benign) from the NLSTx database. The original image data sets for NLSTx are freely available through the NLST dataset [14].

## II. Materials and Methods

**Fig. 2.**
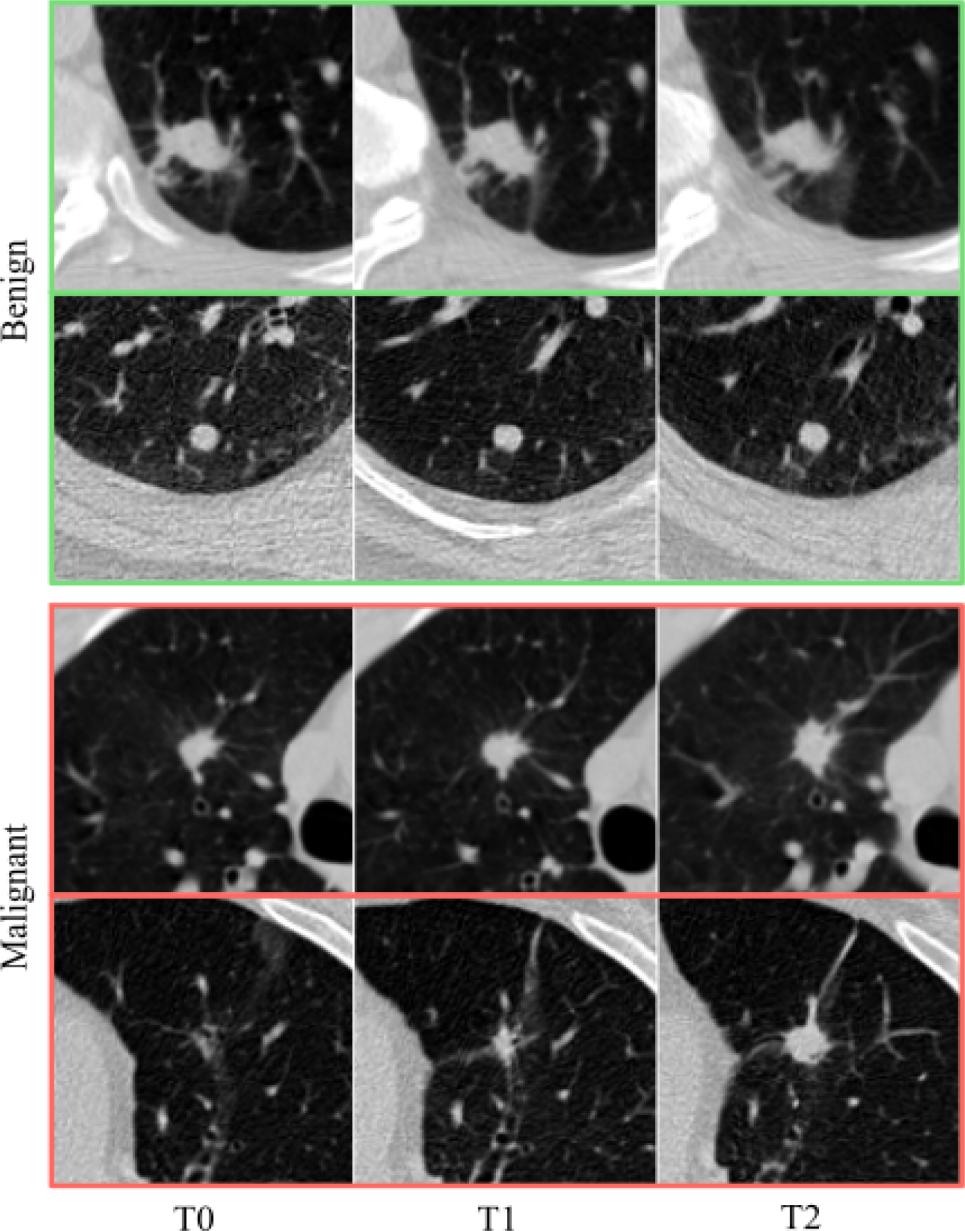
Benign (green) and malignant (red) nodules from the NLSTx dataset across three time points, one year apart (T0–T2).

The NLSTx dataset served as the basis for tuning and evaluating our methods. It consists of 857 biopsy-confirmed lung nodules from individual subjects, of which 207 are classified as malignant. Each nodule is tracked through annual imaging over a three-year period, resulting in a comprehensive collection of nearly 2400 training samples. These CT scans feature slice thicknesses less than 2.5 mm, averaging 2.32 mm, and in-plane resolutions ranging from 0.48 to 0.89 mm, with an average of 0.66 mm. We standardized the image intensities to a resolution of (0.67, 0.67, 2) mm per voxel using the normalization methods described in references [17], [18]. Fig. 2 presents a selection of images from the NLSTx dataset, center-sliced at the nodules’ centroids for optimal visualization.

To further validate our methodology, we extended our experimentation to the Lung Image Database Consortium image collection (LIDC) [1]. Although distinct in composition, the LIDC dataset, served as a complementary resource for evaluation. We filtered out scans with slice thicknesses exceeding 2.5 mm and selected nodules annotated by at least three radiologists with a median malignancy score of either less than or greater than 3 on a scale of 1 to 5 (where 5 indicates a high likelihood of malignancy). The resulting dataset comprised 647 nodules, of which 384 were benign and 263 malignant. Unlike NLSTx, the LIDC nodules are not biopsy-confirmed for malignancy and do not include longitudinal imaging. However, the LIDC's rigorous radiologist-based annotation process and its diverse nodule characteristics provide a robust platform for validating our approach.

**Fig. 3.**
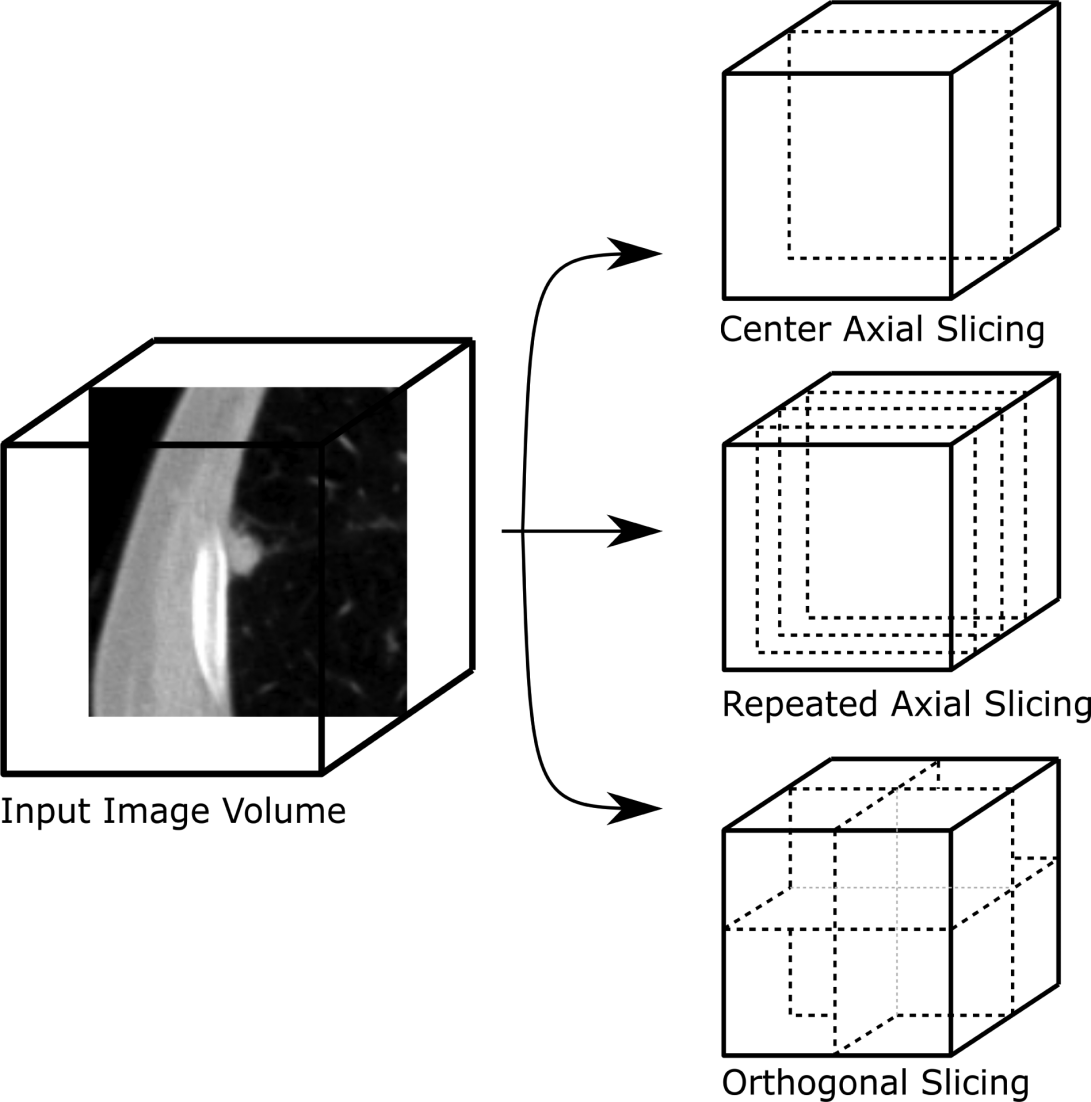
Illustrations of the different input configurations compared in this work. Orthogonal slicing was found to result in better classification performance due to the large quantity of 3D information that it captures.

To evaluate domain adaptation of LVMs to competing methods and prior studies, we designed an experimental setup that allowed for varying specific modeling variables while keeping others constant. This approach focused on the effect of different input image configurations (Fig. 3), input crop sizes of equal lengths along each axis, fine-tuning/adaptation techniques, and model architectures (Table I), all the while maintaining consistency in the data and validation technique. In total, we trained 324 model variants, representing combinations of 3 input configurations, 4 crop sizes, 4 adaptation techniques, and 9 models for each dataset. The subsequent sections provide further details.

**TABLE I:** Overview of Large Vision Models Used

**Name**	**Arch.**	**SSL Method**	**Params**
DINOv2-s	ViT	DINOv2 [13]	22M
DINOv2-b	ViT	DINOv2 [13]	86.5M
DINOv2-l	ViT	DINOv2 [13]	304M
DINOv2-g	ViT	DINOv2 [13]	1.1B
SwinV2-s	Swin	SimMIM [11]	49M
SwinV2-b	Swin	SimMIM [11]	86.9M
SwinV2-l	Swin	SimMIM [11]	195M
ConvNext-V2-b	CNN	MCMAE [21]	87.7M
ConvNext-V2-l	CNN	MCMAE [21]	196M

Overview of the pretrained models used in this work. “-s” = small, “-b” = base, “-l” = large, “-g” = giant, and Arch. = Network Architecture.

### A. Input Image Configurations

Adapting the 2D LVMs for 3D data required creation of input images that capture the context of the nodules. By extracting three orthogonal slices - mid-axial, mid-sagittal, and mid-coronal - from the center of each nodule within the NLSTx and LIDC datasets - and assigning them to the three input channels, our aim was to retain a substantial amount of the pertinent 3D information. In this format the information could also be fed to the 2D LVMs. To evaluate the significance of this 3D context, a single center-axial slice was also used as a baseline technique where the slice was duplicated three times for the three channels to ensure compatibility with 2D LVMs. Additionally, this approach was assessed against a repeated axial slicing method, wherein 3 central and consecutive slices were independently sampled, and fed to 3 independent channels. Fig. 3 demonstrates each of these input sampling methodologies on an image volume.

### B. Domain Adaptation Methods

**Fig. 4.**
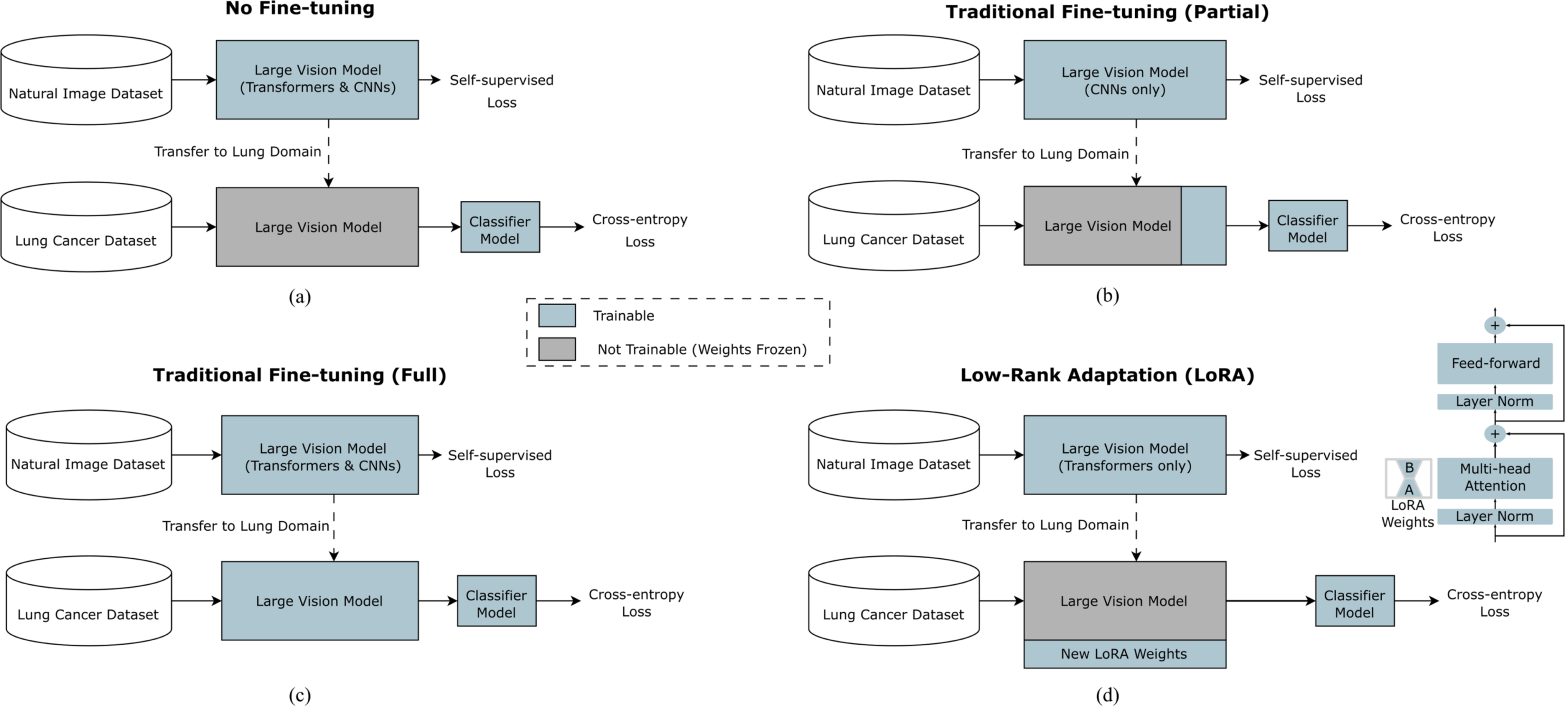
Overview of domain adaptation strategies evaluated. With no fine-tuning (a) only a classifier model's weights are optimized on top of the LVM's extracted image features. Fine-tuning differentiates into two types: partial (b) and full (c), where later network layers are tuned in partial fine-tuning and all layers are tuned in full fine-tuning. Partial fine-tuning is the de facto approach for adapting CNNs while full fine-tuning is for transformers. LoRA (d) [7] is a recent approach for transformers where the pretrained transformer is frozen and a small set of weights are typically added to multi-head attention layers within the transformer which, once trained, adapt the overall model to the new domain without affecting the base model's weights. The right-hand side of (d), a modified vision transformer block is shown with newly added LoRA matrices, A and B to the multi-head attention layer. Only these matrices in each transformer block are trained during domain adaptation while the original transformer parameters are kept frozen, making training faster and more memory-efficient while being more parameter efficient.

Traditionally, adapting neural network models for image classification has involved fine-tuning the feature extractor weights with a small learning rate while training a new classification head for the new, out-of-domain task. This can be achieved by tuning either the entire feature extractor or only select layers of the model. For CNNs, it has been common practice to fine-tune a few of the last layers for domain adaptation. In contrast, fine-tuning ViTs, typically involves fine-tuning the entire network. This difference arises from the distinct ways CNNs and ViTs represent images: CNNs build hierarchical representations through successive layers, while ViTs learn global representations from the initial layer. LoRA [7] introduces a low-rank adapter matrix to the attention layers of each ViT's transformer block (see Fig. 4(d)) which is typical in language model adaptation. Rather than tuning the original ViT weights, these matrices are adjusted, redefining the attention mechanism weight updates as h = W_0_x+ΔWx = W_0_x+α/r(ABx), where W_0_ ∊ ℝ^dxk^ represents the original weights and A ∊ ℝ^dxr^ and B ∊ ℝ^rxk^ are the new low-rank matrices. The rank, r, is smaller than the original weights, resulting in fewer trainable parameters without sacrificing, and sometimes enhancing, performance compared to traditional methods. The α parameter adjusts the influence of the new matrix outputs relative to the original network's outputs.

In this work, we methodically explore the traditional approaches to domain adaptation along with the newer LoRA method. We defined four adaptation techniques below for comparison along with shortened naming conventions for each that will be used in this paper for brevity:

1) **No Fine-Tuning (“No-FT”)** – A baseline where the LVM parameters are keep frozen, with only the linear classification layer weights trained on lung nodule data.
2) **Partial Traditional Fine-Tuning (“Trad-FT (Partial)”**) – The typical approach for CNNs, fine-tuning a select number of the final layers with a low learning rate.
3) **Full Traditional Fine-Tuning (“Trad-FT (Full)”**) – The established method for transformers, fine-tuning all layers with a low learning rate. This approach can also be performed for CNNs but is often found to be less effective and more computationally costly than partial fine-tuning.
4) **Low Rank Adaptation (“LoRA”)** – The domain adaptation method under examination in this study, aiming for fewer parameters, faster training, and improved classification performance.

Fig. 4 provides a visual comparison of each method that shows how LVMs used in this work were pre-trained on large, natural image domain datasets using SSL losses [10], [12], [20] and then adapted to lung cancer classification.

### C. Large Vision Models

Three classes of LVM architectures were examined in this study to gain a broad understanding of how they perform during domain adaptation for lung cancer classification, namely, DINOv2 ViT [13], SwinV2 transformer [11], and the ConvNext-V2 convolutional network [21]. These LVMs were selected because they are each contender for state-of-the-art and cover a diverse set of architectures from the traditional ViT to the hierarchical Swin transformer to a large convolutional network. To evaluate their robustness to model size, we utilized multiple size variants of each from small to giant. Each model had undergone pretraining using self-supervised learning on large, natural image domain datasets as depicted in Fig. 4. For instance, the original DINOv2, a 1B parameter ViT trained on 142 million natural images, was distilled [6] into smaller architectures after the initial training like the 86.5 million parameter base model [13]. Table I shows a comparison of the different model architectures, detailing their pretraining methods and parameter counts.

For each of these pretrained LVMs, a randomly initialized linear classification layer was appended to the feature extractor before domain adaptation. Performance on both the NLSTx and LIDC datasets for lung malignancy classification was measured as a binary classification task using cross-entropy loss.

### D. Training Parameters

Models underwent 5-fold cross-validation, employing the AdamW optimizer to minimize cross-entropy loss. A class weighting of 2.5:1 (malignant to benign) addressed class imbalance on NLSTx whereas for LIDC we used a weighting of 1.5:1. For LoRA and No-FT scenarios, a learning rate of 1e-3 was used, while a reduced rate of 1e-5 was applied for methods fine-tuning feature extractor parameters. Intensity standardization that aligned with each LVM's original training in the natural image domain was implemented across all methods, alongside random data augmentations, including translations, rotations, and scaling. And finally, early stopping regularization was used to avoid overfitting to the training data.

For LoRA tuning, experimentation was done to find the optimal LoRA parameters by holding the α parameter constant at 16 while the rank, r, was varied between 16 and 48 on a single cross-validation fold of the NLSTx data. Through this, the r value that gave the highest classification scores was determined to be 32 and was, thus, used in all experiments. However, for practical purposes we note that varying r had a low impact on overall performance in our experimentation.

## III. Results

**Fig. 5.**
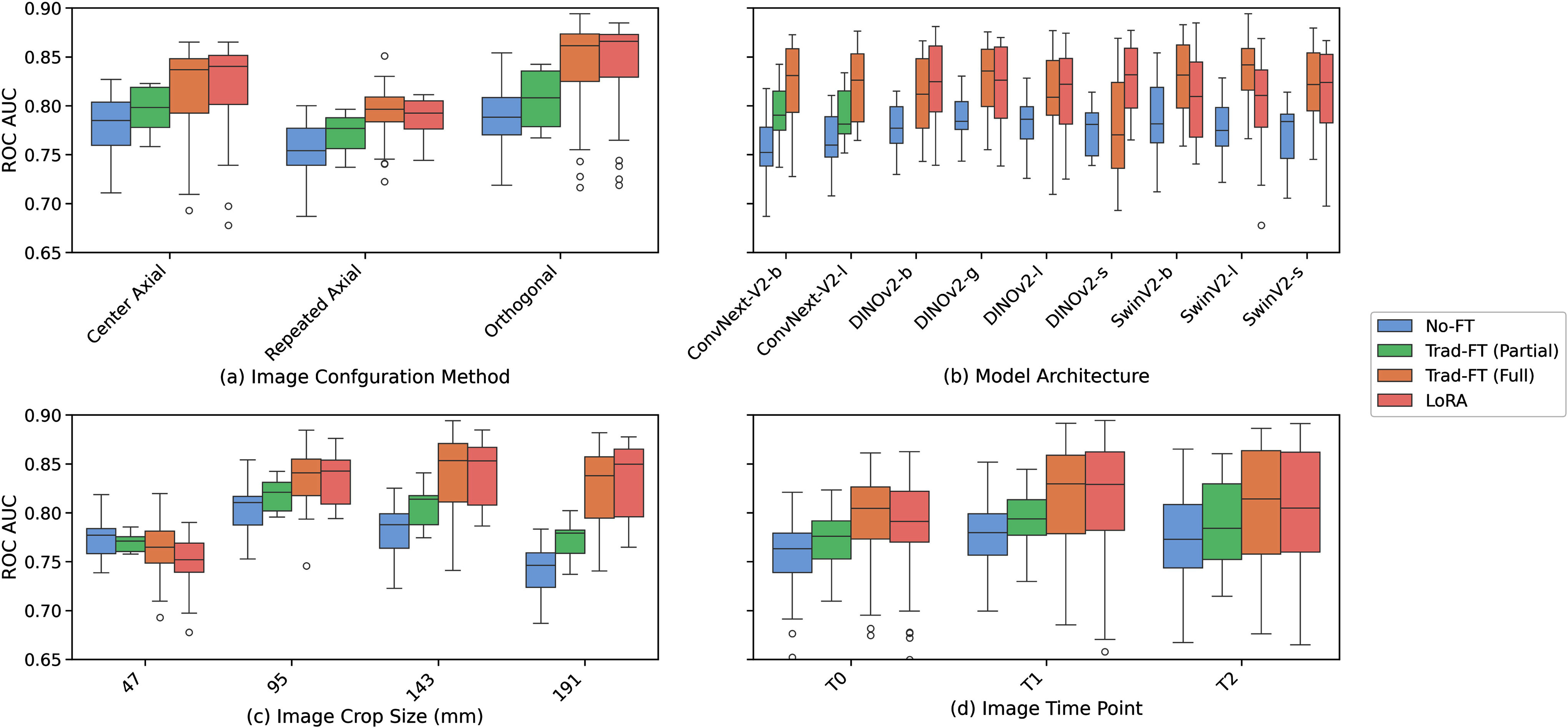
Analysis of the impact of varying the (a) input image configuration, (b) model architecture, (c) image crop size, and (d) NLSTx time point on the domain adaptation methods. Each boxplot represents the distributions of ROC AUC scores for the 324 model configurations (combinations of the 3 input configurations, 4 crop sizes, 4 adaptation techniques, and 9 model architectures) investigated in this work. Each configuration consists of different combinations of these variables (with the ConvNext models only included for the No-FT and Trad-FT (Partial) boxes); the proposed model configuration was selected by choosing the LoRA configuration parameter that achieved the max ROC AUC performance across all variables, which is the SwinV2-b model with 143 mm orthogonal inputs. This model is compared to the state-of-the-art models on NLSTx and LIDC datasets in Table II. We note that (b) clearly shows that, on average, Trad-FT can achieve slightly higher performance on the SwinV2-b and -l models than when using LoRA but will show in Table II that the LoRA models are competitive when are segmented into scan timepoints due to the inherent differences in class distributions for each timepoint. We also considered the other benefits of LoRA in size of parameters and training time efficiency when proposing the final model configuration.

The results of the experimental setup can be described in two parts. The first part is a high-level boxplot analysis of the ROC AUC, shown in Fig. 5, that compares the domain adaptation techniques for the NLSTx data (ref. [18]) across the variables discussed in Section II. To arrive at the results in Table II, we used the boxplot analysis to select the best performing LVMs and compare their performance on the NLSTx and LIDC data. Table II also reports results from applications of other state-of-the-art approaches.

**TABLE II:** Comparison Against Existing Works

**Data**	**Model**	**Tuning**	**Params**	**Train Time**	**ROC AUC**	**F1**	**Sensitivity**	**Specificity**
NLSTx (T0)	EfficientNet [17]	-	43 M	-	0.759	-	-	-
	CAN1-2D [19]	-	14.9 M	-	0.826	-	-	-
	DINOv2-b	No-FT	3074	13.6	0.756±0.035	0.651±0.016	0.643±0.021	0.701±0.053
		Trad-FT	86.5 M	29.3	0.836±0.049	0.736±0.048	0.716±0.045	0.779±0.037
		LoRA	1.2 M	23.3	0.854±0.027	0.732±0.007	0.713±0.019	0.801±0.021
	SwinV2-l	No-FT	3074	48.1	0.798±0.037	0.678±0.067	0.682±0.066	0.865±0.042
		Trad-FT	195 M	122.7	0.854±0.030	0.759±0.018	0.768±0.022	0.893±0.025
		LoRA	2.3 M	101.1	0.819±0.065	0.738±0.061	0.752±0.055	0.875±0.050
	**SwinV2-b**	No-FT	2050	11.7	0.811±0.044	0.680±0.042	0.750±0.038	0.750±0.070
		Trad-FT	86.9 M	26.1	0.853±0.038	0.747±0.032	0.800±0.035	0.831±0.060
		**LoRA**	**1.5 M**	**20.7**	**0.862±0.026**	**0.747±0.035**	**0.802±0.025**	**0.820±0.052**
NLSTx (T1)	EfficientNet [17]	-	43 M	-	0.759	-	-	-
	CAN1-2D [19]	-	14.9 M	-	0.868	-	-	-
	DINOv2-b	No-FT	3074	13.6	0.767±0.035	0.659±0.016	0.653±0.021	0.695±0.060
		Trad-FT	86.5 M	29.3	0.873±0.032	0.774±0.062	0.758±0.074	0.804±0.037
		LoRA	1.2 M	23.3	0.883±0.022	0.774±0.009	0.757±0.017	0.818±0.027
	SwinV2-l	No-FT	3074	48.1	0.781±0.019	0.713±0.035	0.706±0.034	0.881±0.020
		Trad-FT	195 M	122.7	0.876±0.036	0.801±0.029	0.807±0.034	0.892±0.045
		LoRA	2.3 M	101.1	0.864±0.033	0.786±0.040	0.795±0.041	0.878±0.040
	**SwinV2-b**	No-FT	2050	11.7	0.821±0.017	0.686±0.023	0.735±0.021	0.723±0.061
		Trad-FT	86.9 M	26.1	0.883±0.029	0.802±0.019	0.839±0.025	0.840±0.037
		**LoRA**	**1.5 M**	**20.7**	**0.894±0.040**	**0.788±0.029**	**0.825±0.036**	**0.830±0.035**
NLSTx (T2)	EfficientNet [17]	-	43 M	-	0.759	-	-	-
	CAN1-2D [19]	-	14.9 M	-	0.879	-	-	-
	DINOv2-b	No-FT	3074	13.6	0.810±0.035	0.658±0.016	0.651±0.021	0.743±0.073
		Trad-FT	86.5 M	29.3	0.874±0.032	0.742±0.029	0.717±0.022	0.814±0.023
		LoRA	1.2 M	23.3	0.889±0.028	0.740±0.026	0.715±0.022	0.822±0.028
	SwinV2-l	No-FT	3074	48.1	0.827±0.037	0.711±0.067	0.732±0.066	0.868±0.042
		Trad-FT	195 M	122.7	0.886±0.017	0.798±0.035	0.847±0.031	0.881±0.044
		LoRA	2.3 M	101.1	0.875±0.038	0.788±0.057	0.834±0.051	0.878±0.048
	**SwinV2-b**	No-FT	2050	11.7	0.834±0.024	0.671±0.037	0.770±0.036	0.728±0.068
		Trad-FT	86.9 M	26.1	0.883±0.033	0.750±0.037	0.830±0.029	0.821±0.042
		**LoRA**	**1.5 M**	**20.7**	**0.891±0.044**	**0.745±0.056**	**0.820±0.059**	**0.824±0.041**
LIDC	Safta et al. [16]	-	-	-	0.977	-	-	-
	Causey et al. [3]	-	-	-	0.993	-	0.942	-
	CAN1-2D [19]	-	14.9 M	-	0.950	-	-	-
	Dey et al. [4]		-	-	0.955	-	-	-
	DINOv2-b	No-FT	3074	4.7	0.851±0.031	0.747±0.020	0.778±0.025	0.798±0.044
		Trad-FT	86.5 M	10.8	0.978±0.011	0.876±0.018	0.912±0.020	0.881±0.026
		LoRA	1.2 M	6.4	0.968±0.018	0.901±0.015	0.916±0.016	0.928±0.017
	SwinV2-l	No-FT	3074	14.6	0.879±0.020	0.775±0.025	0.788±0.026	0.857±0.024
		Trad-FT	195 M	38.9	0.973±0.015	0.898±0.016	0.921±0.023	0.917±0.023
		LoRA	2.3 M	26.6	0.961±0.017	0.901±0.014	0.915±0.019	0.932±0.026
	**SwinV2-b**	No-FT	2050	4.2	0.891±0.030	0.772±0.024	0.813±0.028	0.824±0.034
		Trad-FT	86.9 M	10.2	0.963±0.38	0.883±0.031	0.905±0.035	0.911±0.041
		**LoRA**	**1.5 M**	**5.8**	**0.970±0.033**	**0.890±0.035**	**0.910±0.027**	**0.915±0.034**

Performance of the best LVMs in this work alongside existing state-of-the-art models for the NLSTx and LIDC datasets. For NLSTx, the dataset was partitioned into the separate time points to show the high gain in performance at the earliest time point, T0, against prior approaches and has clinical importance for early detection and treatment planning. Comparing tuning methods, we see that LoRA consistently achieves competitive performance and does so with low requirements for training (i.e., low trainable parameters and training times). The proposed model is in bold. Training time in sec/epoch.

In Table II, we primarily focus on three classification metrics: number of trainable parameters, training time per epoch, and ROC AUC. However, we also consider additional classification metrics, including the F1 score, sensitivity (recall), and specificity to provide a more comprehensive view of the models’ effectiveness in diagnosing lung cancer.

**Fig. 6.**
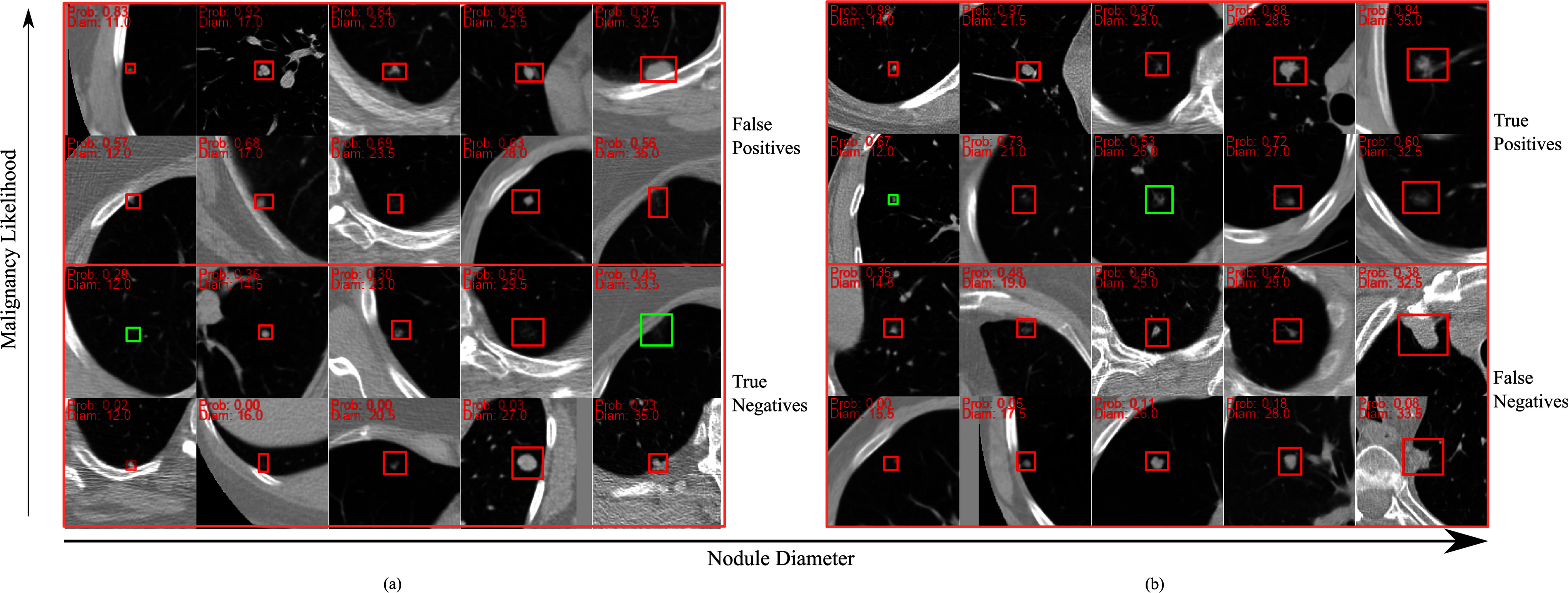
A random sample of benign (a) and malignant (b) nodules, arranged in order of increasing malignancy likelihood and nodule diameter. Each nodule is outlined in red or green, with diameters and model-predicted malignancy likelihood displayed on each image. Nodules are labeled along the righthand side of the figure as being true/false positives and true/false negatives. Green-outlined nodules represent instances where the LoRA model correctly classified the nodule, while the Trad-FT model misclassified it. Specifically, in (a), the green-outlined nodules had trad-FT probabilities of 0.75 and 0.81 (left to right) compared to LoRA predictions of 0.29 and 0.45, indicating a notable improvement (since nodules are indeed benign). Similarly, in (b), green-outlined nodules had Trad-FT probabilities of 0.31 and 0.46 whereas LoRA predictions were 0.53 and 0.67 (since nodules are malignant), again demonstrating a relatively large difference in malignancy predictions.

Qualitative analysis was performed on a random sample of nodules from NLSTx to ensure that the classification metrics used in Table II align with human interpretation. Images for this analysis are shown in Fig. 6.

## IV. Discussion

The results of our experiments confirm the hypothesis of this paper that LoRA adaptation of LVMs is a strong and more efficient and therefore better approach to lung cancer diagnosis than existing approaches. As seen in the results, we were also able to determine a model – using a combination of input configurations, preprocessing, architecture selection, and adaptation method – that outperforms the previous state-of-the-art models on NLSTx at all time points and achieves competitive performance on the well-established LIDC dataset.

### A. Statistical Analysis

The boxplots in Fig. 5 compare the adaptation methods for the 324 model variations explored in this study on NLSTx. This was done to test the hypothesis that parameter efficient fine-tuning can outperform traditional fine-tuning in classification performance. Each plot shown in Fig. 5 examines the variables against the ROC AUC classification performance for all models. At a high-level, we can see that in every group the LVMs that used LoRA adaptation achieved higher ROC AUC performance, on average, except when using a DINOv2-g, SwinV2-l or SwinV2-b architecture, when the crop size was 47 mm, or at the earliest time point (T0) in the NLSTx data, though – in general – was only outperformed by Trad-FT (Full) which is inefficient both in terms of compute-time and number of parameters. We find this to be a motivating result for two reasons. Firstly, that transformer-based LVMs are presenting themselves as viable options for lung cancer classification, and secondly, that LoRA can be used in place of fully fine-tuning a transformer's weights – which opens the door to utilizing large transformer models in the clinical workflow. On a practical note, we also see that larger model variants in Fig. 5(b) do not necessarily result in better performance and, instead, we see performance mostly plateau, especially when using LoRA.

Furthermore, Fig. 5 shows that LoRA and Trad-FT (Full) far outperformed Trad-FT (Partial) for CNNs in nearly every scenario. This suggests that – while LVMs are trained on massive datasets of natural images – the features in the early layers of CNNs are not well-optimized for lung cancer classification and benefit from being further tuned which is efficiently done with LoRA on transformers. This is supported by the orthogonal slicing input results in Fig. 5(a) that show a much larger jump in performance for LoRA and Trad-FT (Full) models when compared to the No-FT and Trad-FT (Partial). We do not find this surprising, as incorporating 3D data as 3 orthogonal slices into the RGB channels of these LVMs is likely far outside the domain of the data they were trained on. These findings, again, should be motivational for researchers interested in utilizing transformer-based LVMs, like DINOv2 or SwinV2, over CNN models to medical imaging classification tasks via LoRA.

Aside from comparing adaptation methods, Fig. 5 additionally allows us to compare different input configurations and input crop sizes that can be selected for the best-performing model variations. The orthogonal slicing input configuration results in higher ROC AUCs for every adaptation technique, leading us to choose this input configuration in our final lung malignancy prediction models. For input crop size, LoRA-based LVMs with 143 mm^3^ input crop achieve the highest classification scores. Interestingly, cases where the early layers of an LVM are not tuned (No-FT and Trad-FT (Partial)), quickly drop in performance as input size increases. This suggests that more aggressive domain adaptation, like Trad-FT (Full) and LoRA, helps stabilize model performance regardless of input size.

Finally, we analyze performance of the models by time point on the NLSTx data. We see a trend where classification performance increases with time. This is unsurprising since the discrepancy between benign and malignant nodules in earlier time points is less apparent making classification more challenging. We also note that the pattern of LoRA adaptation contending with Trad-FT (Full) is seen along each time point, supporting the main hypothesis of this paper.

### B. Top Model Performance Comparison

Using the findings from our statistical analysis, we selected our best-performing model variants to analyze lung malignancy classification performance against prior approaches on the NLSTx and LIDC datasets. More specifically, we chose DINOv2-b, SwinV2-l, and SwinV2-b models that use orthogonal input slicing and 143 mm^3^ input crop sizes. The performance for these models, displayed in Table II, show that the LoRA-adapted variant of Swinv2-b beats prior approaches on NLSTx by 3% ROC AUC while using 89.9% fewer trainable parameters, making this model the new state-of-the-art on NLSTx. It also uses significantly fewer trainable parameters and trains more than 10 seconds faster per epoch than when fully fine-tuning all the network parameters. Similar observations are made for the LIDC results, showing that domain adaptation with LoRA on LVMs is a generalized approach and further supports the paper's initial hypothesis.

Clinically, a 3% gain in ROC-AUC along with comparable sensitivity and specificity to traditional fine-tuning approaches may offer only modest improvements in practice. However, we wish to emphasis the practical advantages of LoRA, particularly its substantial reduction in parameters and the ease of transferability for various clinical applications. By requiring only small LoRA models trained atop pre-trained vision models, LoRA streamlines the engineering effort needed for deployment and maintenance, ultimately reducing the time required to place these tools into clinicians’ hands to improve patient care.

### C. Qualitative Analysis

In addition to statistical analyses, we conducted a human evaluation of individual images and model outputs to ensure that model predictions aligned with visual features. Fig. 6 presents a random sample of nodules arranged by increasing diameter and malignancy likelihood as estimated by our best-performing model, SwinV2-b. Each image is categorized as false positives, true negatives, true positives, or false negatives using a 0.5 classification threshold to assess the model's performance. Visual inspection of true positives and true negatives reveals that benign nodules are often round, solid, and have clear boundaries, while correctly predicted malignant nodules typically have irregular boundaries, lobulated shapes, and are less solid. It is reassuring to see that the model assigns appropriate malignancy likelihoods to larger benign nodules and smaller malignant ones, indicating it has learned diverse features beyond nodule diameter. Analyzing false positives and negatives provides insight into areas for improvement. The false positives include non-solid nodules with ill-defined boundaries, which could reasonably confuse human experts. False negatives, though more consequential, show no single feature bias influencing misclassifications. Notably, the confusion matrix and sensitivity in Table II indicate that our best model produces few false negatives (less than 4%), demonstrating high performance.

### D. Limitations and Future Work

This study has several limitations. While the NLSTx and LIDC datasets provide a solid foundation, a broader range of clinical imaging data could add valuable diversity. Additionally, although models pre-trained on natural images were used here due to the limited availability of extensive medical imaging datasets (as discussed in the Introduction), future studies could explore models pre-trained on specialized medical imaging data when such data becomes available. Such models would likely have better classification performance, as they would learn features unique to medical images. There is also intriguing potential to investigate large language models (LLMs) for simulating diagnostic classifiers [10]. Though current LLMs are not expected to be extensively trained on medical data, it would be interesting to begin evaluating their performance against models like those used in this work.

## V. Conclusion

This study has demonstrated that the remarkable progress achieved in the natural image classification domain with large vision models holds significant potential for improving lung malignancy classification. We have shown that LoRA (Low-Rank Adaptation) is particularly effective in tailoring a variety of large vision models (LVMs) for the task of malignancy classification. With PEFT and LoRA, we have achieved superior classification performance to existing 3D techniques while using only partial 3D information (2.5D). This enhancement comes with the added benefits of a substantial reduction in the number of trainable parameters and faster training speeds per epoch. These findings underscore the value of LoRA in bridging the gap between large-scale, pretrained vision models and their application in the lung cancer diagnostics. The success of this approach in lung cancer classification also underscores the broader potential of LoRA and large transformer models to revolutionize the medical imaging field. By building on the achievements of large self-supervised pretrained transformers in the natural image analysis, these models could drive significant advances in the medical image interpretation and diagnosis tasks.

## VI. Authors Contributions

Conceptualization and project design, B.V. and A. A. Methodology and Experimental Design, B.V. and A. A. Software and Validation, B.V. Writing—original draft preparation, B.V. Writing—review, and editing, B.V. and A. A. Both authors have read and agreed with the published version of the manuscript.

## VII. Conflict of Interest

The authors declare no conflicts of interest in the current work.
